# B-REAL: A keypoint-less behavior platform for offline classification and real-time detection

**DOI:** 10.64898/2026.10.13.758637

**Published:** 2026-10-20

**Authors:** A Eagle, B Kohler, V Kamrowski, N Lingam, R Li, EA Yttri

## Abstract

Two obstacles limit automated behavior analysis in neuroethology: the burden of first training pose estimation models, and the inability to identify behavior quickly enough for real-time classification that could trigger stimuli, drug delivery, or environmental manipulations. Here we present B-REAL, a pose-free classifier that maps raw video to behavior faster than video frames are recorded, even on a CPU. Given substantially less training data, B-REAL matched or surpassed established methods trained on full benchmark rodent or fly datasets, particularly on rare and even single-frame behaviors. Pretraining on unlabeled video further improves its accuracy without slowing inference or training time. In closed loop, rewarding mice for B-REAL-detected grooming more than doubled their performance of grooming bouts. B-REAL thus makes closed-loop behavioral experiments practical with minimal annotation and standard hardware.

## Full Text Availability

The license terms selected by the author(s) for this preprint version do not permit archiving in PMC. The full text is available from the preprint server.

